# Supplementary material for: Higher ventricular rate during atrial fibrillation relates to increased cerebral hypoperfusions and hypertensive events
Source: Sci Rep. 2019 Mar 7;9:3779. doi: 10.1038/s41598-019-40445-5 (PMC6405756; doi:10.1038/s41598-019-40445-5)
Supplement: Supplementary file 1 — Supplementary Information [file 41598_2019_40445_MOESM1_ESM.pdf]

Supplementary Information

Higher ventricular rate during atrial fibrillation relates to increased cerebral hypoperfusion and hypertensive events

Saglietto A., Scarsoglio S., Ridolfi L., Gaita F., Anselmino M.

**NSR**					
	50 bpm	70 bpm	90 bpm	110 bpm	130 bpm
RR [s]: $\mu \pm \sigma$ (cv=0.07)	1,20 ± 0,08	0,86 ± 0,06	0,67 ± 0,05	0,55 ± 0,04	0,46 ± 0,03
**AF**					
	50 bpm	70 bpm	90 bpm	110 bpm	130 bpm
RR [s]: $\mu \pm \sigma$	1,20 ± 0,29	0,86 ± 0,21	0,67 ± 0,16	0,55 ± 0,13	0,46 ± 0,11
γ [Hz]	3,56	6,71	8,47	9,58	10,35
(cv=0.24)					

Supplementary Table 1: Mean (μ) and standard deviation (σ) values of the RR beating during NSR (cv=0.07) and AF (cv=0.24) at the different HRs considered. The rate parameter, γ, is also reported for the AF cases.

	70-50 bpm	70-90 bpm	70-110 bpm	70-130 bpm
$\overline{Q}_{ICA,left}$	$1{,}48 \cdot 10^{-17}$	$6{,}61 \cdot 10^{-14}$	$< 10^{-20}$	$< 10^{-20}$
$\overline{Q}_{MCA,left}$	$3{,}06 \cdot 10^{-15}$	$3{,}43 \cdot 10^{-16}$	$< 10^{-20}$	$< 10^{-20}$
$\overline{Q}_{dm,left}$	$1{,}63 \cdot 10^{-10}$	$4{,}07 \cdot 10^{-16}$	$< 10^{-20}$	$< 10^{-20}$
$\overline{Q}_{pv}$	$7{,}74 \cdot 10^{-14}$	$1{,}22 \cdot 10^{-16}$	$< 10^{-20}$	$< 10^{-20}$
$\overline{P}_a$	$< 10^{-20}$	$< 10^{-20}$	$< 10^{-20}$	$< 10^{-20}$
$\overline{P}_{MCA,left}$	$< 10^{-20}$	$< 10^{-20}$	$< 10^{-20}$	$< 10^{-20}$
$\overline{P}_{dm,left}$	$< 10^{-20}$	$< 10^{-20}$	$< 10^{-20}$	$< 10^{-20}$
$\overline{P}_c$	$1,48 \cdot 10^{-17}$	$2,22 \cdot 10^{-19}$	$< 10^{-20}$	$< 10^{-20}$

Supplementary Table 2: **p-values of the Kolmogorov-Smirnov test (5% significance level) for the distributions of the mean flow rate per beat ($\overline{Q}$) and mean pressure per beat ($\overline{P}$) during AF.** The reference distribution is taken at 70 bpm, and the test is performed for each average hemodynamic variable comparing the following AF simulations: 70-50 bpm, 70-90 bpm, 70-110 bpm, 70-130 bpm. When p-values are below 10^{-20}, the notation $< 10^{-20}$ is adopted.

Cardiovascular model

The cardiovascular model proposed by [2, 6, 7] is coupled to a baroreceptor model [4] to describe the whole (arterial and venous) circulation system in presence of short-term autoregulation. The model is composed by a network of compliances, C, resistances, R, and inductances, L, representing the pumping heart coupled to the systemic and pulmonary systems. The viscous effects are taken into account by the resistances, R [mmHg s/ml], the inertial terms are considered by the inductances, L [mmHg s^2/ml], while the elastic vessel properties are described by the compliances, C [ml/mmHg]. Three cardiovascular variables are involved at each section: the blood flow, Q [ml/s], the volume, V [ml], the pressure, P [mmHg].

All the four chambers of the pumping heart are described as active. The pulsatility properties are included by means of two pairs of time-varying elastance functions, one for the atria and one for the ventricles, which are then used in the constitutive equations (relating pressure, P, and volume, V). For the four heart valves, the basic pressure-flow relation is described by an orifice model. The valve motion mechanisms are deeply analyzed and account for the following blood-flow effects: pressure difference across the valve, frictional effects from neighboring tissue resistance, the dynamic motion effect of the blood acting on the valve leaflet, the action of the vortex downstream of the valve. An equation for the mass conservation (accounting for the volume variation, dV/dt) concludes the description of each chamber.

The systemic and pulmonary systems are divided into five sections. The systemic arteries are described by four sections (aortic sinus, artery, arteriole and capillary), while the systemic venous circulation is characterized by a unique compartment. The pulmonary circuit follows the same architecture. Each section of the systemic and pulmonary circuits may contain three components (the viscous term, R, the inertial term, L, and the elastic term, C), and is characterized by three equations: an equation of motion (accounting for the flow variation, dQ/dt), an equation for the conservation of mass (expressed in terms of pressure variations, dP/dt), and a linear state equation between pressure and volume.

The baroreceptor model combines the afferent and the central nervous system into a unified description, so that the sympathetic, n_s, and parasympathetic, n_p, activities are directly related to the

carotid sinus pressure through a steady state sigmoidal function. The efferent responses consist of a static component, which is a linear combination of n_s and n_p, and a dynamic component, which is a first order ordinary differential equation in time. The short-term baroregulation accounts for the inotropic effect of both ventricles (neglecting for simplicity the parasympathetic influence), as well as the control of the systemic vasculature (peripheral arterial resistances, unstressed volume of the venous system, and venous compliance). The chronotropic effects due to the heart rate regulation, which differently act during NSR and AF, are instead implicitly and intrinsically taken into account by the RR extraction.

Equations

For the sake of simplicity, the equations are grouped into cardiac (with the four chambers), circulatory (with systemic and pulmonary loops), and baroreceptor mechanism sections.

Left atrium

$$\begin{aligned} \frac{dV_{la}}{dt} &= Q_{pvn} - Q_{mi}, \\ P_{la} &= P_{la,un} + E_{la}(V_{la} - V_{la,un}), \\ Q_{mi} &= \begin{cases} CQ_{mi} AR_{mi} \sqrt{P_{la} - P_{lv}}, & \text{if } P_{la} \geq P_{lv}, \\ -CQ_{mi} AR_{mi} \sqrt{P_{lv} - P_{la}}, & \text{if } P_{la} < P_{lv}, \end{cases} \end{aligned} \tag{S1}$$

where the subscript *un* denotes the unstressed pressure and volume levels of each cardiovascular section. The time-varying elastance is

$$E_{la}(t) = E_{la,min} + \frac{E_{la,max} - E_{la,min}}{2} e_a(t), \tag{S2}$$

and the atrium activation function is

$$e_a(t) = \begin{cases} 0, & \text{if } 0 \leq t \leq T_{ac}, \\ 1 - \cos\left(\frac{t - T_{ac}}{RR - T_{ac}} 2\pi\right), & \text{if } T_{ac} < RR, \end{cases} \tag{S3}$$

where $E_{la,min}$ and $E_{la,max}$ are the minimum and maximum elastance values, respectively, while T_{ac} is the beginning of atrial contraction.

The valve opening is decided by the angular position of the leaflets:

$$AR_{mi} = \frac{(1 - \cos\theta_{mi})^2}{(1 - \cos\theta_{max})^2} \tag{S4}$$

and the valve motion is governed by

$$\frac{d^2\theta_{mi}}{dt^2} = \begin{cases} (P_{la} - P_{lv})K_{p,mi}\cos\theta_{mi} - K_{f,mi}\frac{d\theta_{mi}}{dt} \\ +K_{b,mi}Q_{mi}\cos\theta_{mi} \\ -K_{v,mi}Q_{mi}\sin 2\theta_{mi}, \quad \text{if} \;\; Q_{mi} \geq 0, \\ (P_{la} - P_{lv})K_{p,mi}\cos\theta_{mi} - K_{f,mi}\frac{d\theta_{mi}}{dt} \\ +K_{b,mi}Q_{mi}\cos\theta_{mi}, \quad \text{if} \;\; Q_{mi} < 0, \end{cases} \tag{S5}$$

Left ventricle

$$\begin{aligned} \frac{dV_{lv}}{dt} &= Q_{mi} - Q_{ao}, \\ P_{lv} &= P_{lv,un} + E_{lv}(V_{lv} - V_{lv,un}), \\ Q_{ao} &= \begin{cases} CQ_{ao}AR_{ao}\sqrt{P_{lv} - P_a}, & \text{if} \;\; P_{lv} \geq P_a, \\ -CQ_{ao}AR_{ao}\sqrt{P_a - P_{lv}}, & \text{if} \;\; P_{lv} < P_a, \end{cases} \end{aligned} \tag{S6}$$

The time-varying elastance is

$$E_{lv}(t) = E_{lv,min} + \frac{E_{lv,max} - E_{lv,min}}{2}e_v(t), \tag{S7}$$

and the ventricle activation function is

$$e_v(t) = \begin{cases} 1-\cos\left(\frac{t}{T_{me}}\pi\right), & \text{if } 0 \le t < T_{me}, \\ 1+\cos\left(\frac{t-T_{me}}{T_{ce}-T_{me}}\pi\right), & \text{if } T_{me} \le t < T_{ce} \\ 0, & \text{if } T_{ce} \le t < RR, \end{cases} \tag{S8}$$

where $E_{lv,min}$ and $E_{lv,max}$ are the minimum and maximum elastance values, respectively. T_{me} and T_{ce} are the instants where the elastance reaches its maximum and constant values, respectively.

The valve opening is decided by the angular position of the leaflets:

$$AR_{ao} = \frac{(1-\cos\theta_{ao})^2}{(1-\cos\theta_{max})^2} \tag{S9}$$

and the valve motion is governed by

$$\frac{d^2\theta_{ao}}{dt^2} = \begin{cases} (P_{lv}-P_a)K_{p,ao}\cos\theta_{ao} - K_{f,ao}\frac{d\theta_{ao}}{dt} \\ +K_{b,ao}Q_{ao}\cos\theta_{ao} \\ -K_{v,ao}Q_{ao}\sin 2\theta_{ao}, & \text{if } Q_{ao} \ge 0, \\ (P_{lv}-P_a)K_{p,ao}\cos\theta_{ao} - K_{f,ao}\frac{d\theta_{ao}}{dt} \\ +K_{b,ao}Q_{ao}\cos\theta_{ao}, & \text{if } Q_{ao} < 0, \end{cases} \tag{S10}$$

Right atrium

$$\begin{aligned} \frac{dV_{ra}}{dt} &= Q_{svn} - Q_{ti}, \\ P_{ra} &= P_{ra,un} + E_{ra}(V_{ra} - V_{ra,un}), \\ Q_{ti} &= \begin{cases} CQ_{ti}AR_{ti}\sqrt{P_{ra}-P_{rv}}, & \text{if } P_{ra} \ge P_{rv}, \\ -CQ_{ti}AR_{ti}\sqrt{P_{rv}-P_{ra}}, & \text{if } P_{ra} < P_{rv}, \end{cases} \end{aligned} \tag{S11}$$

The time-varying elastance is

$$E_{ra}(t) = E_{ra,min} + \frac{E_{ra,max} - E_{ra,min}}{2} e_a(t), \tag{S12}$$

where the activation function is given by Eq. (S3), while $E_{ra,min}$ and $E_{ra,max}$ are the minimum and maximum elastance values, respectively.

The valve opening is decided by the angular position of the leaflets:

$$AR_{ti} = \frac{(1 - \cos\theta_{ti})^2}{(1 - \cos\theta_{max})^2} \tag{S13}$$

and the valve motion is governed by

$$\frac{d^2\theta_{ti}}{dt^2} = \begin{cases} (P_{ra} - P_{rv})K_{p,ti}\cos\theta_{ti} - K_{f,ti}\frac{d\theta_{ti}}{dt} \\ +K_{b,ti}Q_{ti}\cos\theta_{ti} \\ -K_{v,ti}Q_{ti}\sin 2\theta_{ti}, \quad \text{if } Q_{ti} \geq 0, \\ (P_{ra} - P_{rv})K_{p,ti}\cos\theta_{ti} - K_{f,ti}\frac{d\theta_{ti}}{dt} \\ +K_{b,ti}Q_{ti}\cos\theta_{ti}, \quad \text{if } Q_{ti} < 0, \end{cases} \tag{S14}$$

Right ventricle

$$\begin{aligned} \frac{dV_{rv}}{dt} &= Q_{ti} - Q_{po}, \\ P_{rv} &= P_{rv,un} + E_{rv}(V_{rv} - V_{rv,un}), \\ Q_{po} &= \begin{cases} CQ_{po}AR_{po}\sqrt{P_{rv} - P_{pas}}, & \text{if } P_{rv} \geq P_{pas}, \\ -CQ_{po}AR_{po}\sqrt{P_{pas} - P_{rv}}, & \text{if } P_{pas} > P_{rv}, \end{cases} \end{aligned} \tag{S15}$$

The time-varying elastance is

$$E_{rv}(t) = E_{rv,min} + \frac{E_{rv,max} - E_{rv,min}}{2} e_v(t), \tag{S16}$$

where the ventricle activation function is given by Eq. (S8), while $E_{rv,min}$ and $E_{rv,max}$ are the minimum and maximum elastance values, respectively.

The valve opening is decided by the angular position of the leaflets:

$$AR_{po} = \frac{(1 - \cos\theta_{po})^2}{(1 - \cos\theta_{max})^2} \tag{S17}$$

and the valve motion is governed by

$$\frac{d^2\theta_{po}}{dt^2} = \begin{cases} (P_{rv} - P_{pas})K_{p,po}\cos\theta_{po} - K_{f,po}\frac{d\theta_{po}}{dt} \\ +K_{b,po}Q_{po}\cos\theta_{po} \\ -K_{v,po}Q_{po}\sin 2\theta_{po}, \quad \text{if } \ Q_{po} \geq 0, \\ (P_{rv} - P_{pas})K_{p,po}\cos\theta_{po} - K_{f,po}\frac{d\theta_{po}}{dt} \\ +K_{b,po}Q_{po}\cos\theta_{po}, \quad \text{if } \ Q_{po} < 0, \end{cases} \tag{S18}$$

Systemic Circuit

$$\begin{cases} \frac{dP_a}{dt} = \frac{Q_{ao}-Q_{sas}}{C_{sas}}, \\ \frac{dQ_{sas}}{dt} = \frac{P_a-P_{sat}-R_{sas}Q_{sas}}{L_{sas}}, \\ P_a - P_{sas,un} = \frac{1}{C_{sas}}(V_{sas} - V_{sas,un}), \end{cases} \tag{S19}$$

$$\begin{cases} \frac{dP_{sat}}{dt} = \frac{Q_{sas}-Q_{sat}}{C_{sat}}, \\ \frac{dQ_{sat}}{dt} = \frac{P_{sat}-P_{svn}-(R_{sat}+R_{sar}+R_{scp})Q_{sat}}{L_{sat}}, \\ P_{sat} - P_{sat,un} = \frac{1}{C_{sat}}(V_{sat} - V_{sat,un}), \end{cases} \tag{S20}$$

$$\begin{cases} \frac{dP_{svn}}{dt} = \frac{Q_{sat}-Q_{svn}}{C_{svn}} - \frac{1}{C_{svn}}\frac{dV_{svn,un}}{dt}, \\ Q_{svn} = \frac{P_{svn}-P_{ra}}{R_{svn}}, \\ P_{svn} - P_{svn,un} = \frac{1}{C_{svn}}(V_{svn} - V_{svn,un}), \end{cases} \tag{S21}$$

Pulmonary Circuit

$$\begin{cases} \frac{dP_{pas}}{dt} = \frac{Q_{po}-Q_{pas}}{C_{pas}}, \\ \frac{dQ_{pas}}{dt} = \frac{P_{pas}-P_{pat}-R_{pas}Q_{pas}}{L_{pas}}, \\ P_{pas} - P_{pas,un} = \frac{1}{C_{pas}}(V_{pas} - V_{pas,un}) \end{cases} \tag{S22}$$

$$\begin{cases} \frac{dP_{pat}}{dt} = \frac{Q_{pas}-Q_{pat}}{C_{pat}}, \\ \frac{dQ_{pat}}{dt} = \frac{P_{pat}-P_{pvn}-(R_{pat}+R_{par}+R_{pcp})Q_{pat}}{L_{pat}}, \\ P_{pat} - P_{pat,un} = \frac{1}{C_{pat}}(V_{pat} - V_{pat,un}), \end{cases} \tag{S23}$$

$$\begin{cases} \frac{dP_{pvn}}{dt} = \frac{Q_{pat}-Q_{pvn}}{C_{pvn}}, \\ Q_{pvn} = \frac{P_{pvn}-P_{la}}{R_{pvn}}, \\ P_{pvn} - P_{pvn,un} = \frac{1}{C_{pvn}}(V_{pvn} - V_{pvn,un}). \end{cases} \tag{S24}$$

Baroreceptor Mechanism Equations

$$\begin{cases} \frac{dE_{lv,max}}{dt} = \frac{1}{\tau_h}\left(-E_{lv,max} + \alpha_{E_{lv}} n_s + \gamma_{E_{lv}}\right), \\ \frac{dE_{rv,max}}{dt} = \frac{1}{\tau_h}\left(-E_{rv,max} + \alpha_{E_{rv}} n_s + \gamma_{E_{rv}}\right), \end{cases} \tag{S25}$$

$$\begin{cases} \frac{dR_{sar}}{dt} = \frac{1}{\tau_a}\left(-R_{sar} + \alpha_{R_{sar}} n_s + \gamma_{R_{sar}}\right), \\ \frac{dR_{scp}}{dt} = \frac{1}{\tau_a}\left(-R_{scp} + \alpha_{R_{scp}} n_s + \gamma_{R_{scp}}\right), \end{cases} \tag{S26}$$

$$\begin{cases} \frac{dC_{svn}}{dt} = \frac{1}{\tau_v}\left(-C_{svn} + \alpha_{C_{svn}} n_s + \gamma_{C_{svn}}\right), \\ \frac{dV_{svn,un}}{dt} = \frac{1}{\tau_v}\left(-V_{svn,un} + \alpha_{V_{svn}} n_s + \gamma_{V_{svn}}\right). \end{cases} \tag{S27}$$

The sympathetic, n_s, and parasympathetic, n_p, activities [Hz] are expressed as

$$\begin{cases} n_s(\overline{P}_{cs}) = \frac{1}{1+\left(\frac{\overline{P}_{cs}}{\mu}\right)^{\nu}}, \\ n_p(\overline{P}_{cs}) = \frac{1}{1+\left(\frac{\overline{P}_{cs}}{\mu}\right)^{-\nu}}, \end{cases} \tag{S28}$$

where $\overline{P}_{cs}$ is the carotid sinus pressure averaged over one cardiac cycle, which is here taken equal to systemic arterial pressure averaged per beat, $\overline{P}_a$ [mmHg s]. μ [mmHg] is the mean systemic arterial pressure in physiological conditions at steady state for HR=70 bpm, while ν is a dimensionless parameter characterizing the steepness of the curves.

Initial Conditions and Cardiovascular Parameters

Initial conditions, which are given in terms of pressures, volumes, flow rates, valve opening angles and hemodynamic variables involved in the baroreceptor mechanisms, are the same for both the normal sinus rhythm and the atrial fibrillation simulations. The total volume is taken as the typical value for a healthy adult, here V_{tot}=5246.42 ml. Flow rates and valve angles are initially considered as all the valves are closed and no flow is present. Initial pressures in the circulatory sections are chosen in the range reached during a normal cardiac cycle. Volumes in the four chambers are obtained at t=0 subtracting from the total volume, V_{tot}, all the volume contributes at the generic vascular section i, using the constitutive relation $P_{i,t=0} - P_{i,un} = \frac{1}{C_i}(V_{i,t=0} - V_{i,un})$, where $P_{i,t=0}$ is imposed as previously said. Table SM 3 summarizes the adopted initial values. Cardiovascular parameters of the model are as well recalled in Tables from SM 4 to SM 9.

Cerebral Model

The lumped model, proposed by Ursino and Giannessi [8], extends the Windkessel approach to the arterial and venous cerebral circulation, and is divided into three main parts: large arteries, distal arterial circulation, and capillary-venous circulation. The model is composed by a network of compliances, C, and resistances, R. The viscous effects are taken into account by the resistances, R [mmHg s/ml], while the elastic vessel properties are described by the compliances, C [ml/mmHg]. Three cardiovascular variables are involved at each section: the blood flow, Q [ml/s], the volume, V [ml], the

pressure, P [mmHg].

Equations

For the sake of simplicity, the equations are grouped following the three main partitions of the model. The autoregulation and CO_2 activity equations for the distal district are separately reported.

Large arteries

$$\begin{cases} C_{ICA,left}\left(\frac{dP_{MCA,left}}{dt} - \frac{dP_{ic}}{dt}\right) = Q_{ICA,left} + Q_{PCoA,left} - Q_{MCA,left} - Q_{ACA1,left}, \\ C_{ICA,right}\left(\frac{dP_{MCA,right}}{dt} - \frac{dP_{ic}}{dt}\right) = Q_{ICA,right} + Q_{PCoA,right} - Q_{MCA,right} - Q_{ACA1,right}, \\ C_{BA}\frac{dP_{BA,willis}}{dt} = Q_{BA} - Q_{PCA1,left} - Q_{PCA1,right}, \end{cases} \tag{S29}$$

$$\begin{cases} Q_{ACA2,left} = Q_{ACA1,left} + Q_{ACoA}, \\ Q_{ACA2,right} = Q_{ACA1,right} - Q_{ACoA}, \\ Q_{PCA2,left} = -Q_{PCoA,left} + Q_{PCA1,left}, \\ Q_{PCA2,right} = -Q_{PCoA,right} + Q_{PCA1,right}, \end{cases} \tag{S30}$$

where the flow rates, Q, are:

$$\begin{cases} Q_{ICA,left} = \frac{P_a - P_{MCA,left}}{R_{ICA,left}}, \\ Q_{ICA,right} = \frac{P_a - P_{MCA,right}}{R_{ICA,right}}, \\ Q_{BA} = \frac{P_a - P_{BA,willis}}{R_{BA}}, \end{cases} \tag{S31}$$

$$\begin{cases} Q_{MCA,left} = \frac{P_{MCA,left} - P_{dm,left}}{R_{MCA,left} + R_{dm,left}/2}, \\ Q_{MCA,right} = \frac{P_{MCA,right} - P_{dm,right}}{R_{MCA,right} + R_{dm,right}/2}, \\ Q_{ACA1,left} = \frac{P_{ICA,left} - P_{ACA,left}}{R_{ACA1,left}}, \\ Q_{ACA1,right} = \frac{P_{ICA,right} - P_{ACA,right}}{R_{ACA1,right}}, \\ Q_{PCA1,left} = \frac{P_{BA,willis} - P_{PCA,left}}{R_{PCA1,left}}, \\ Q_{PCA1,right} = \frac{P_{BA,willis} - P_{PCA,right}}{R_{PCA1,right}}, \end{cases} \tag{S32}$$

$$
\begin{cases}
Q_{ACA2,left} = \dfrac{P_{ACA,left} - P_{da,left}}{R_{ACA2,left} + R_{da,left}/2}, \\
Q_{ACA2,right} = \dfrac{P_{ACA,right} - P_{da,right}}{R_{ACA2,right} + R_{da,right}/2}, \\
Q_{ACoA} = \dfrac{P_{ACA,right} - P_{ACA,left}}{R_{ACoA}}, \\
Q_{PCA2,left} = \dfrac{P_{PCA,left} - P_{dp,left}}{R_{PCA2,left} + R_{dp,left}/2}, \\
Q_{PCA2,right} = \dfrac{P_{PCA,right} - P_{dp,right}}{R_{PCA2,right} + R_{dp,right}/2}, \\
Q_{PCoA,left} = \dfrac{P_{PCA,left} - P_{MCA,left}}{R_{PCoA,left}}, \\
Q_{PCoA,right} = \dfrac{P_{PCA,right} - P_{MCA,right}}{R_{PCoA,right}},
\end{cases} \tag{S33}
$$

Distal arterial circulation

$$
\begin{cases}
\dfrac{dV_{dm,left}}{dt} = Q_{MCA,left} - Q_{dm,left} + Q_{cam,left} + Q_{cpm,left}, \\
\dfrac{dV_{dm,right}}{dt} = Q_{MCA,right} - Q_{dm,right} + Q_{cam,right} + Q_{cpm,right}, \\
\dfrac{dV_{da,left}}{dt} = Q_{ACA2,left} - Q_{da,left} - Q_{cam,left} + Q_{caa}, \\
\dfrac{dV_{da,right}}{dt} = Q_{ACA2,right} - Q_{da,right} - Q_{cam,right} - Q_{caa}, \\
\dfrac{dV_{dp,left}}{dt} = Q_{PCA2,left} - Q_{dp,left} - Q_{cpm,left} + Q_{cpp}, \\
\dfrac{dV_{dp,right}}{dt} = Q_{PCA2,right} - Q_{dp,right} - Q_{cpm,right} - Q_{cpp},
\end{cases} \tag{S34}
$$

where the flow rates, Q, are:

$$
\begin{cases}
Q_{dm,left} = \dfrac{P_{dm,left} - P_c}{R_{dm,left}/2}, \\
Q_{dm,right} = \dfrac{P_{dm,right} - P_c}{R_{dm,right}/2}, \\
Q_{da,left} = \dfrac{P_{da,left} - P_c}{R_{da,left}/2}, \\
Q_{da,right} = \dfrac{P_{da,right} - P_c}{R_{da,right}/2}, \\
Q_{dp,left} = \dfrac{P_{dp,left} - P_c}{R_{dp,left}/2}, \\
Q_{dp,right} = \dfrac{P_{dp,right} - P_c}{R_{dp,right}/2},
\end{cases} \tag{S35}
$$

$$
\begin{cases}
Q_{cam,left} = \dfrac{P_{da,left} - P_{dm,left}}{R_{cam,left}}, \\[2ex]
Q_{cam,right} = \dfrac{P_{da,right} - P_{dm,right}}{R_{cam,right}}, \\[2ex]
Q_{cpm,left} = \dfrac{P_{dp,left} - P_{dm,left}}{R_{cpm,left}}, \\[2ex]
Q_{cpm,right} = \dfrac{P_{dp,right} - P_{dm,right}}{R_{cpm,right}}, \\[2ex]
Q_{caa} = \dfrac{P_{da,right} - P_{da,left}}{R_{caa}}, \\[2ex]
Q_{cpp} = \dfrac{P_{dp,right} - P_{dp,left}}{R_{cpp}},
\end{cases}
\tag{S36}
$$

while the constitutive relations are:

$$
\begin{cases}
P_{dm,left} = \dfrac{V_{dm,left}}{C_{dm,left}} + P_{ic}, \\[2ex]
P_{dm,right} = \dfrac{V_{dm,right}}{C_{dm,right}} + P_{ic}, \\[2ex]
P_{da,left} = \dfrac{V_{da,left}}{C_{da,left}} + P_{ic}, \\[2ex]
P_{da,right} = \dfrac{V_{da,right}}{C_{da,right}} + P_{ic}, \\[2ex]
P_{dp,left} = \dfrac{V_{dp,left}}{C_{dp,left}} + P_{ic}, \\[2ex]
P_{dp,right} = \dfrac{V_{dp,right}}{C_{dp,right}} + P_{ic},
\end{cases}
\tag{S37}
$$

Capillary-venous circulation

$$
\begin{cases}
C_{ic}\dfrac{dP_{ic}}{dt} = Q_{MCA,left} - Q_{dm,left} + Q_{PCA2,left} - Q_{dp,left} + Q_{ACA2,left} - Q_{da,left} \\[2ex]
\qquad + Q_{MCA,right} - Q_{dm,right} + Q_{PCA2,right} - Q_{dp,right} + Q_{ACA2,right} - Q_{da,right} \\[2ex]
\qquad + Q_{pv} - Q_{vs} + Q_f - Q_o, \\[2ex]
C_{vi}\left(\dfrac{dP_v}{dt} - \dfrac{dP_{ic}}{dt}\right) = Q_{pv} - Q_{vs}, \\[2ex]
Q_{dm,left} + Q_{da,left} + Q_{dp,left} + Q_{dm,right} + Q_{da,right} + Q_{dp,right} = Q_f + Q_{pv},
\end{cases}
\tag{S38}
$$

where the flow rates, Q, the compliances C_{ic}, C_{vi}, and the resistance R_{vs} are defined as follows:

$$
\begin{cases}
Q_{pv} = \dfrac{P_c - P_v}{R_{pv}}, \\
Q_{vs} = \dfrac{P_v - P_{vs}}{R_{vs}}, \\
Q_f = \begin{cases} \dfrac{P_c - P_{ic}}{R_f}, & \text{if } P_c \geq P_{ic}, \\ 0, & \text{if } P_c < P_{ic}, \end{cases} \\
Q_o = \begin{cases} \dfrac{P_{ic} - P_{vs}}{R_o}, & \text{if } P_{ic} \geq P_{vs}, \\ 0, & \text{if } P_{ic} < P_{vs}, \end{cases} \\
Q_{out} = Q_{vs} + Q_o,
\end{cases}
\tag{S39}
$$

$$
\begin{cases}
C_{ic} = \dfrac{1}{k_E P_{ic}}, \\
C_{vi} = \dfrac{1}{k_{ven}(P_v - P_{ic} - P_{v1})}, \\
R_{vs} = \begin{cases} \dfrac{P_v - P_{vs}}{P_v - P_{ic}} R_{vs1}, & \text{if } P_{vs} < P_{ic}, \\ R_{vs1}, & \text{if } P_{vs} \geq P_{ic}, \end{cases}
\end{cases}
\tag{S40}
$$

Autoregulation and CO_2 reactivity equations

For each of the six distal regions, the following equations hold:

$$
\begin{cases}
\tau_{aut} \dfrac{dx_{aut,i,j}}{dt} = -x_{aut,i,j} + G_{aut} \left(\dfrac{Q_{di,j} - Q_{ndi,j}}{Q_{ndi,j}} \right), \quad \text{i=m,a,p; j=left,right}, \\
\tau_{CO_2} \dfrac{dx_{CO_2,i,j}}{dt} = -x_{CO_2,i,j} + G_{CO_2} A_{CO_2,i,j} \log_{10} \left(\dfrac{P_{aCO_2}}{P_{aCO_2 n}} \right), \quad \text{i=m,a,p; j=left,right},
\end{cases}
\tag{S41}
$$

where the subscript n denotes the basal values and

$$
A_{CO_2,i,j} = \frac{1}{1 + \exp\{[-k_{CO_2}(Q_{di,j} - Q_{ndi,j})/Q_{ndi,j}] - b_{CO_2}\}}, \quad \text{i=m,a,p; j=left,right.} \tag{S42}
$$

Distal compliances and resistances are ruled by the following relations:

$$
\begin{cases}
C_{di,j} = \dfrac{C_{d0i,j}\left[(1-\Delta C_{di,j}/2) + (1+\Delta C_{di,j}/2)\exp\left(\frac{x_{CO_2,i,j}-x_{aut,i,j}}{k_{C_{di,j}}}\right)\right]}{1+\exp[(x_{CO_2,i,j}-x_{aut,i,j})/k_{C_{di,j}}]}, \\
R_{di,j} = \dfrac{k_{R_{di,j}} C_{d0i,j}^2}{V_{di,j}^2}, \quad \text{i=m,a,p; j=left,right,}
\end{cases} \tag{S43}
$$

with

$$
\Delta C_{di,j} = \begin{cases} 2s_1, & \text{if } x_{CO_2,i,j} < x_{aut,i,j}, \\ 2s_2, & \text{if } x_{CO_2,i,j} \geq x_{aut,i,j}, \end{cases} \quad \text{i=m,a,p; j=left,right}
$$

$$
k_{C_{di,j}} = \begin{cases} \dfrac{C_{d0i,j}s_1}{2}, & \text{if } x_{CO_2,i,j} < x_{aut,i,j}, \\ \dfrac{C_{d0i,j}s_2}{2}, & \text{if } x_{CO_2,i,j} \geq x_{aut,i,j}, \end{cases} \quad \text{i=m,a,p; j=left,right} \tag{S44}
$$

Initial Conditions and Cerebral Parameters

Initial conditions are given in terms of pressures, volumes, autoregulation and CO_2 reactivity state variables, and are equal in both NSR and AF conditions. The initial setting is chosen according to flow rate repartition given by [8] with the following total basal values: basal cerebral blood flow $Q_n = 12.5$ ml/s, basal distal resistance $R_{dn} = 5.4$ mmHg s/ml, basal distal compliance $C_{dn} = 0.2$ ml/mmHg. State variables for autoregulation, x_{aut}, and CO_2 reactivity, x_{CO_2}, are initially considered as no activity is present. Initial pressures in the cerebral regions are chosen in the physiological range reached during a normal cardiac cycle. Volumes in the distal region j are obtained at t=0 using the constitutive relation $V_{dj,t=0} = C_{dj,t=0}(P_{dj,t=0} - P_{ic,t=0})$, where $P_{dj,t=0}$ and $P_{ic,t=0}$ are imposed as previously said. The adopted initial conditions are listed in Table SM 10, while parameters of the cerebral model are reported in Tables from SM 11 to SM 14.

Numerical Scheme

The differential equations of both models are solved by means of a multistep adaptive solver, implemented by the `ode15s` Matlab function. This variable order solver is based on the numerical differentiation formulas (NDFs) and is chosen because is one of the most efficient and suitable routines for stiff problems. In fact, the differential system of both models shows some stiffness features, that

is the equations include some terms that can lead to rapid variation in the solutions. This aspect is particularly relevant during end-diastolic and end-systolic phases, when the valves opening and closure cause sudden variations of the leaflet angular position.

References

[1] Guyton AC, Hall JE (2006) Textbook of Medical Physiology. Elsevier Saunders, pp 1116

[2] Korakianitis T, Shi Y (2006) Numerical simulation of cardiovascular dynamics with healthy and diseased heart valves. J Biomech 39: 1964-1982

[3] Korakianitis T, Shi Y (2006) A concentrated parameter model for the human cardiovascular system including heart valve dynamics and atrioventricular interaction. Med Eng Phys 28: 613628

[4] Ottesen JT, Olufsen MS, Larsen JK (2004) Applied Mathematical Models in Human Physiology. SIAM, pp 298

[5] Ruiz P, Amin Rezaienia M, Rahideh A, Keeble TR, Rothman MT, Korakianitis T (2013) In vitro cardiovascular system emulator (bioreactor) for the simulation of normal and diseased conditions with and without mechanical circulatory support. Artif Organs 37: 549-560

[6] Scarsoglio S, Guala A, Camporeale C, Ridolfi L (2014) Impact of atrial fibrillation on the cardiovascular system through a lumped-parameter approach. Med Biol Eng Comput 52: 905-920

[7] Scarsoglio S, Camporeale C, Guala A, Ridolfi L (2016) Fluid dynamics of heart valves during atrial fibrillation: a lumped parameter-based approach. Comput Methods Biomech Biomed Engin 10(19): 1060-1068

[8] Ursino M, Giannessi M (2010) A model of cerebrovascular reactivity including the circle of willis and cortical anastomoses. Ann Biomed Eng 38: 955-974

[9] Westerhof N, Stergiopulos N, Noble MIM (2010) Snapshots of Hemodynamics. Springer, pp 286

Variable	Value ($t = 0$)
$V_{la,0}$	60 ml
$V_{lv,0}$	130.06 ml
$V_{ra,0}$	39 ml
$V_{rv,0}$	110 ml
$P_{sas,0}$	124.75 mmHg
$Q_{sas,0}$	0 ml/s
$P_{sat,0}$	124.75 mmHg
$Q_{sat,0}$	0 ml/s
$P_{svn,0}$	10 mmHg
$P_{pas,0}$	21.11 mmHg
$Q_{pas,0}$	0 ml/s
$P_{pat,0}$	21.11 mmHg
$Q_{pat,0}$	0 ml/s
$P_{pvn,0}$	10 mmHg
$\theta_{mi,0} = d\theta_{mi,0}/dt$	0 rad
$\theta_{ao,0} = d\theta_{ao,0}/dt$	0 rad
$\theta_{ti,0} = d\theta_{ti,0}/dt$	0 rad
$\theta_{po,0} = d\theta_{po,0}/dt$	0 rad
$E_{lv,max,0}$	2.5 mmHg/ml
$E_{rv,max,0}$	1.15 mmHg/ml
$R_{sar,0}$	0.44 mmHg s/ml
$R_{scp,0}$	0.4576 mmHg s/ml
$C_{svn,0}$	20.5 ml/mmHg
$V_{svn,un,0}$	3000 ml

Supplementary Table 3: Cardiovascular model: initial conditions.

Parameter	Value
CQ_{ao}	350 ml/(s mmHg$^{0.5}$)
CQ_{mi}	400 ml/(s mmHg$^{0.5}$)
$E_{lv,min}$	0.07 mmHg/ml
$P_{lv,un}$	1 mmHg
$V_{lv,un}$	5 ml
$E_{la,max}$	0.25 mmHg/ml
$E_{la,min}$	0.15 mmHg/ml
$P_{la,un}$	1 mmHg
$V_{la,un}$	4 ml
CQ_{po}	350 ml/(s mmHg$^{0.5}$)
CQ_{ti}	400 ml/(s mmHg$^{0.5}$)
$E_{rv,min}$	0.07 mmHg/ml
$P_{rv,un}$	1 mmHg
$V_{rv,un}$	10 ml
$E_{ra,max}$	0.25 mmHg/ml
$E_{ra,min}$	0.15 mmHg/ml
$P_{ra,un}$	1 mmHg
$V_{ra,un}$	4 ml

Supplementary Table 4: Cardiovascular model: heart parameters.

Parameter	Value
C_{sas}	0.064 ml/mmHg
R_{sas}	0.003 mmHg s/ml
L_{sas}	0.000062 mmHg s^2/ml
$P_{sas,un}$	1 mmHg
$V_{sas,un}$	25 ml
C_{sat}	1.28 ml/mmHg
R_{sat}	0.05 mmHg s/ml
L_{sat}	0.0017 mmHg s^2/ml
$P_{sat,un}$	1 mmHg
$V_{sat,un}$	775 ml
R_{svn}	0.075 mmHg s/ml
$P_{svn,un}$	1 mmHg

Supplementary Table 5: Cardiovascular model: systemic circulation parameters.

Parameter	Value
C_{pas}	0.162 ml/mmHg
R_{pas}	0.002 mmHg s/ml
L_{pas}	0.000052 mmHg s^2/ml
$P_{pas,un}$	1 mmHg
$V_{pas,un}$	25 ml
C_{pat}	3.42 ml/mmHg
R_{pat}	0.01 mmHg s/ml
L_{pat}	0.0017 mmHg s^2/ml
$P_{pat,un}$	1 mmHg
$V_{pat,un}$	175 ml
R_{par}	0.0275 mmHg s/ml
R_{pcp}	0.0385 mmHg s/ml
R_{pvn}	0.006 mmHg s/ml
C_{pvn}	20.5 ml/mmHg
$P_{pvn,un}$	1 mmHg
$V_{pvn,un}$	300 ml

Supplementary Table 6: Cardiovascular model: pulmonary circulation parameters.

Parameter	Value
$K_{p,mi}$, $K_{p,ao}$, $K_{p,ti}$, $K_{p,po}$	5500 ml/mmHg
$K_{f,mi}$, $K_{f,ao}$, $K_{f,ti}$, $K_{f,po}$	50 s^{-1}
$K_{b,mi}$, $K_{b,ao}$, $K_{b,ti}$, $K_{b,po}$	2 rad/(s ml)
$K_{v,mi}$, $K_{v,ti}$	3.5 rad/(s ml)
$K_{v,ao}$, $K_{v,po}$	7 rad/(s ml)
θ_{max}	5/12 π rad

Supplementary Table 7: Cardiovascular model: valve dynamics parameters.

Parameter	Value
T_{ac}	0.875 RR s
T_{me}	0.3 $\sqrt{RR}$ s
T_{ce}	3/2 T_{me} s

Supplementary Table 8: Cardiovascular model: temporal parameters. Activation times are those typically introduced in the time-varying elastance models [2–5], and are here settled considering that, at RR=0.8 s, the ventricular systole lasts about 0.3 s, while the atrial systole length is about 0.1 s [1].

Parameter	Value
τ_h	3 s
τ_a	12 s
τ_v	60 s
μ	97.13 mmHg
ν	7
$\alpha_{E_{lv}}$	1 mmHg s/ml
$\gamma_{E_{lv}}$	2 mmHg/ml
$\alpha_{E_{rv}}$	0.46 mmHg s/ml
$\gamma_{E_{rv}}$	0.92 mmHg/ml
$\alpha_{R_{sar}}$	0.3520 mmHg s^2/ml
$\gamma_{R_{sar}}$	0.2640 mmHg s/ml
$\alpha_{R_{scp}}$	0.3660 mmHg s^2/ml
$\gamma_{R_{scp}}$	0.2746 mmHg s/ml
$\alpha_{C_{svn}}$	-4.1 ml s/mmHg
$\gamma_{C_{svn}}$	22.55 ml/mmHg
$\alpha_{V_{svn}}$	-1260 ml s
$\gamma_{V_{svn}}$	3630 ml

Supplementary Table 9: Cardiovascular model: baroreceptor parameters. Time delays (τ_h: heart, τ_a: arterial, τ_v: vein) and coefficients (α_i, γ_i, μ, ν) for the strength of the sympathetic and parasympathetic activities are taken according to the validated model by [4].

Variable	Value ($t = 0$)
$P_{ic,0}$	9.5 mmHg
$P_{v,0}$	14 mmHg
$P_{dm,left,0}$	58.5 mmHg
$V_{dm,left,0}$	2.94 ml
$P_{dm,right,0}$	58.5 mmHg
$V_{dm,right,0}$	2.94 ml
$P_{da,left,0}$	58.5 mmHg
$V_{da,left,0}$	0.78 ml
$P_{da,right,0}$	58.5 mmHg
$V_{da,right,0}$	0.78 ml
$P_{dp,left,0}$	58.5 mmHg
$V_{dp,left,0}$	1.18 ml
$P_{dp,right,0}$	58.5 mmHg
$V_{dp,right,0}$	1.18 ml
$P_{ICA,left,0}$	100 mmHg
$P_{ICA,right,0}$	100 mmHg
$P_{BA,willis,0}$	100 mmHg
$x_{aut,i,j,0}$ i=m,a,p, j=left,right	0
$x_{CO_2,i,j,0}$ i=m,a,p, j=left,right	0

Supplementary Table 10: Cerebral model: initial conditions.

Parameter	Value
$R_{ICA,left}$	0.5689 mmHg s/ml
$R_{ICA,right}$	0.5689 mmHg s/ml
R_{BA}	0.4501 mmHg s/ml
$R_{MCA,left}$	1.4419 mmHg s/ml
$R_{MCA,right}$	1.4419 mmHg s/ml
$R_{PCA1,left}$	0.7640 mmHg s/ml
$R_{PCA1,right}$	0.7640 mmHg s/ml
$R_{ACA1,left}$	3.7912 mmHg s/ml
$R_{ACA1,right}$	3.7912 mmHg s/ml
$R_{PCA2,left}$	3.6063 mmHg s/ml
$R_{PCA2,right}$	3.6063 mmHg s/ml
$R_{ACA2,left}$	1.6227 mmHg s/ml
$R_{ACA2,right}$	1.6227 mmHg s/ml
$R_{PCoA,left}$	90.9786 mmHg s/ml
$R_{PCoA,right}$	90.9786 mmHg s/ml
R_{ACoA}	14.9228 mmHg s/ml
$C_{ICA,left}$	0.0034 ml/mmHg
$C_{ICA,right}$	0.0034 ml/mmHg
C_{BA}	0.0017 ml/mmHg

Supplementary Table 11: Cerebral model: large arteries parameters.

Parameter	Value
$R_{cam,left}$	105 mmHg s/ml
$R_{cam,right}$	105 mmHg s/ml
$R_{cpm,left}$	120 mmHg s/ml
$R_{cpm,right}$	120 mmHg s/ml
R_{caa}	22 mmHg s/ml
R_{cpp}	75 mmHg s/ml

Supplementary Table 12: Cerebral model: distal arterial circulation parameters.

Parameter	Value
R_f	$2.3 \cdot 10^3$ mmHg s/ml
R_o	526.3 mmHg s/ml
R_{pv}	0.880 mmHg s/ml
R_{vs1}	0.366 mmHg s/ml
k_E	0.077 ml^{-1}
k_{ven}	0.155 ml^{-1}
P_{vs}	6 mmHg
P_{v1}	-2.5 mmHg

Supplementary Table 13: Cerebral model: capillary-venous circulation parameters.

Parameter	Value
τ_{aut}	20 s
G_{aut}	0.9
τ_{CO_2}	40 s
G_{CO_2}	4.0
k_{CO_2}	15
b_{CO_2}	0.5
P_{aCO_2n}	40 mmHg
P_{aCO_2}	40 mmHg
s_1	7
s_2	0.4
$Q_{ndm,left}$	3.75 ml/s
$Q_{ndm,right}$	3.75 ml/s
$Q_{nda,left}$	1 ml/s
$Q_{nda,right}$	1 ml/s
$Q_{ndp,left}$	1.5 ml/s
$Q_{ndp,right}$	1.5 ml/s
$k_{R_{dm,left}}$	$4.2848 \cdot 10^{4}$ mmHg^{-3} s/ml
$k_{R_{dm,right}}$	$4.2848 \cdot 10^{4}$ mmHg^{-3} s/ml
$k_{R_{da,left}}$	$1.6060 \cdot 10^{5}$ mmHg^{-3} s/ml
$k_{R_{da,right}}$	$1.6060 \cdot 10^{5}$ mmHg^{-3} s/ml
$k_{R_{dp,left}}$	$1.0777 \cdot 10^{5}$ mmHg^{-3} s/ml
$k_{R_{dp,right}}$	$1.0777 \cdot 10^{5}$ mmHg^{-3} s/ml
$C_{d0m,left}$	0.06 ml/mmHg
$C_{d0m,right}$	0.06 ml/mmHg
$C_{d0a,left}$	0.016 ml/mmHg
$C_{d0a,right}$	0.016 ml/mmHg
$C_{d0p,left}$	0.024 ml/mmHg
$C_{d0p,right}$	0.024 ml/mmHg

Supplementary Table 14: Cerebral model: autoregulation and CO_2 reactivity parameters.
